# Reduced functional connectivity of somatosensory network in writer's cramp patients

**DOI:** 10.1002/brb3.433

**Published:** 2016-01-26

**Authors:** Chia‐Hsiung Cheng, Yi‐Jhan Tseng, Rou‐Shayn Chen, Yung‐Yang Lin

**Affiliations:** ^1^ Department of Occupational TherapyGraduate Institute of Behavioral SciencesChang Gung UniversityTaoyuanTaiwan; ^2^ Healthy Aging Research CenterChang Gung UniversityTaoyuanTaiwan; ^3^ Department of PsychiatryChang Gung Memorial HospitalTaoyuanTaiwan; ^4^ Institute of PhysiologyNational Yang‐Ming UniversityTaipeiTaiwan; ^5^ Laboratory of NeurophysiologyTaipei Veterans General HospitalTaipeiTaiwan; ^6^ Department of NeurologyChang Gung Memorial HospitalTaoyuanTaiwan; ^7^ College of MedicineChang Gung UniversityTaoyuanTaiwan; ^8^ Institute of Brain ScienceNational Yang‐Ming UniversityTaipeiTaiwan; ^9^ Department of NeurologyTaipei Veterans General HospitalTaipeiTaiwan

**Keywords:** Coherence, dystonia, magnetoencephalography (MEG), median nerve stimulation, secondary somatosensory cortex (SII)

## Abstract

**Background:**

The involvement of motor cortex and sensorimotor integration in patients with writer's cramp (WC) has been well documented. However, the exact neurophysiological profile within the somatosensory system, including primary somatosensory cortex (SI), contralateral (SIIc), and ipsilateral (SIIi) secondary somatosensory areas remains less understood.

**Methods:**

This study investigated the neuromagnetic cortical activities of median nerve stimulation in 10 patients with WC and 10 healthy controls (HC). To comprehensively explore all the aspects of somatosensory functioning, we analyzed our data with the minimum norm estimate (MNE), the time‐frequency approach with evoked and induced activities, and functional connectivity between SI and SIIc (SI–SIIc), SI and SIIi (SI–SIIi), and SIIc and SIIi (SIIc–SIIi) from theta to gamma oscillations.

**Results:**

No significant between‐group differences were found in the MNE cortical amplitudes of SI, SIIc, and SIIi. Power strengths of evoked gamma oscillation and induced beta synchronization were also equivalent between WC and HC groups. However, we found significantly reduced theta coherence of SI–SIIi, alpha coherence of SI–SIIi and SIIc–SIIi, as well as beta coherence of SIIc–SIIi in patients with WC.

**Conclusion:**

Our results suggest the involvement of somatosensory abnormalities, primarily with the form of functional connectivity, in patients with WC.

## Introduction

Patients with focal hand dystonia experience task‐specific involuntary movements due to abnormal cocontraction of agonist and antagonist muscles. In writer's cramp (WC), a type of hand dystonia, a growing number of neurophysiological and imaging studies have indicated that motor cortical dysfunction or sensorimotor integration impairment is the core manifestation of this disease (Berardelli et al. 1998; Quartarone et al. 2003; Tecchio et al. 2006, 2008; Hallett 2011). However, the exact pathophysiology of somatosensory processes has not been fully clarified.

Various forms of somatosensory dysfunction have been reported in patients with WC. For example, the distance of cortical representation of digits was smaller in WC, suggesting a disorganization of the primary somatosensory cortex (SI) (Bara‐Jimenez et al. 1998; Tinazzi et al. 2003). Increased somatosensory temporal discrimination threshold (STDT) in patients with focal hand dystonia was also reported, either in the affected or unaffected hand (Scontrini et al. 2009; Conte et al. 2014). By using electrophysiological recordings in conjunction with dual electrical stimulation in temporal pattern, the neural correlates of STDT deficits have been observed and found to be correlated with the STDT severity (Frasson et al. 2001; Tamura et al. 2008). When dual electrical stimulation was applied in the distinct locations, the results indicated an abnormality in inhibitory integration of afferent inputs coming from adjacent body parts (Tinazzi et al. 2000). Furthermore, sensory trick, a phenomenon of transient alleviation of dystonic symptoms by means of sensory stimulation, highlights the critical role of somatosensory processing in the pathophysiology of clinical manifestation. Nevertheless, it remains debatable whether the registration and identification of the sensory information in the somatosensory cortex is impaired in patients with WC. Data from electrophysiological research demonstrated inconsistent findings, with some studies reporting abnormal somatosensory‐evoked potentials (SEPs) or fields (SEFs) of SI (Tamura et al. 2008; Dolberg et al. 2011), whereas others showing relatively preserved function of SEPs or SEFs (Cimatti et al. 2007; Tseng et al. 2014) in WC or other forms of focal hand dystonia. Most importantly, the exact profile of secondary somatosensory (SII) cortical responses, which are relevant to higher order somatic processes, also remains elusive.

In addition to time‐domain measurement, time‐frequency analysis provides additional information and solves the questions that SEPs or SEFs could not answer. The evoked oscillations reflect time‐ and phase‐locked synchronization of neural assemblies. Compared to the alpha and beta frequency bands, the somatosensory‐evoked gamma oscillations (above 30 Hz) represent the short‐latency, early‐stage information processing of the human SI. Although it has been reported that the power of evoked gamma oscillations was comparable between healthy subjects and patients with WC (Cimatti et al. 2007), the relevant evidence remains scarce. As for the induced oscillations, event‐related synchronization (ERS) of beta frequency bands (13–30 Hz) occurs around 500 to 700 msec after the stimulus onset, and has been widely shown to be located in SI (Pfurtscheller et al. 2001; Cheyne et al. 2003), whereas others have indicated in sensorimotor areas (Hari et al. 1998; Gaetz and Cheyne 2006). This neurophysiological component is proposed to subserve an inhibition of motor cortex (Salmelin et al. 1995) or a sensory reafference (Cassim et al. 2001). One previous study (Tseng et al. 2014) did not find significant differences in beta ERS strength in SI between normal participants and WC at the sensor‐level analysis. A precise characteristic of beta ERS at the source level is warranted. It is also striking to note that cortical somatosensory information is processed not only at SI and SII individually, but also at a network across relevant areas. Cortico‐cortical coherence is commonly taken as a measurement of firing synchronization of two different neural assemblies. However, up to the present, it remains poorly understood whether the interplay of SI and SII in WC is affected.

To address the aforementioned issues, this study investigated the somatosensory information processes in WC by acquiring magnetoencephalographic (MEG) data during electrical stimulation to the median nerve at rest. We firstly aimed to verify the previous SEP or SEF findings at the cortical level. The distributed source imaging technique of minimum norm estimate (MNE) was applied to detect the source activity in the SI, contralateral SII (SIIc), and ipsilateral SII (SIIi). Then we tested whether abnormalities of evoked gamma oscillation and induced beta ERS in SI were present in patients with WC. Most importantly and notably, our major goal was to examine the cortical coherence between SI and SIIc (SI–SIIc), SI and SIIi (SI–SIIi) as well as SIIc and SIIi (SIIc–SIIi). It was hypothesized that patients with WC exhibited a reduced functional connectivity compared to healthy controls.

## Methods

### Subjects

Ten right‐handed patients with WC (five males and five females; 19–59 years old) and ten age‐, gender‐, and handedness‐matched healthy controls (HC, five males and five females; 22–56 years old) participated in this study. Majority of subjects were from an earlier study (Tseng et al. 2014). Patients with idiopathic WC were diagnosed by an experienced neurologist (RS Chen) and were free from medication for at least 14 days prior to MEG recordings. Among these patients, only one received injection of botulinum toxin (BTX) 3 month prior to the current MEG recordings. This protocol was reviewed and approved by the Institutional Review Board of Taipei Veterans General Hospital.

### Somatosensory stimulation and MEG recordings

Each participant received 0.2‐msec electrical stimulation of the right median nerve, with a constant interstimulus interval (ISI) of 3 sec. The stimulus intensity was set 20% above the motor threshold to elicit a visible twitch of the thumb (Cheng and Lin 2013). During the recordings, the subjects sat comfortably and were asked to relax and fix their eyes on a centrally presented crosshair.

The magnetic responses were recorded with a 306‐channel MEG instrument (Vectorview, Elekta‐Neuromag, Helsinki, Finland). The online bandpass filter and sampling rate were set to [0.1, 160] Hz and 500 Hz, respectively. Responses contaminated by eye blinks (electro‐oculogram >150 *μ*V) were discarded. In each participant, at least 100 artifact‐free trials were obtained for source analysis of SEFs.

### Source analysis of SEFs

The averaged SEF data were offline filtered with a bandpass of [0.1, 120] Hz and analyzed with an epoch of 500 msec, including a 100‐msec prestimulus baseline.

The modeling of the cortical spatiotemporal dynamics of SEFs was obtained with Brainstorm software (Tadel et al. 2011). An overlapping‐sphere model was applied as the forward modeling of MEG measures. The cortically constrained source imaging of depth‐weighted minimum norm estimate (MNE) was utilized to create the individual source map, and then geometrically rescaled to the Montreal Neurological Institute brain template (Colin27; Cheng et al. 2013, 2015a).

The identification of regions of interest (ROIs) was based on the empirical evidence that electrical stimulation to the median nerve elicits cortical activation of SI (M20 and M35), SIIc, and SIIi (Lin et al. 2000; Lin and Forss 2002; Cheng et al. 2015b). A cluster of 30 vertices corresponding to 3–4 cm^2^ was manually selected to define each ROI. The time‐resolved magnitude of each elementary source was normalized to its fluctuations over baseline, yielding a set of *z* score at each cortical location. The *z*‐score values were rectified to detect absolute magnitude changes above baseline level.

### Time‐frequency analysis

In order to characterize the power spectrum of somatosensory cortical responses, MEG source waveforms of each raw trial (100 msec before and 1000 msec after stimulus onset) were transformed using Morlet wavelet‐based time‐frequency decomposition (central frequency: 1 Hz; time resolution: 3 sec) as in Brainstorm software. Since SI demonstrates a higher signal‐to‐noise ratio for both evoked and induced responses, the time‐frequency investigation was focused on this specific ROI. The power of signal fluctuations was computed between 1 and 50 Hz in 1‐Hz steps.

The mean strength of evoked gamma oscillation (30–50 Hz) was calculated from the average of 15–45 msec after stimulus onset. For the induced beta ERS, we selected peak amplitudes of the most reactive frequency bands (2 Hz) from each participant. For both evoked and induced spectral power data, the *z* score was again utilized to detect the absolute value with respect to the baseline power level.

### Functional connectivity analysis

Each raw data of time‐frequency response (−100 to 1000 msec) were further analyzed for brain dynamic activities. Source‐based coherence among SI, SIIc, and SIIi was estimated by using magnitude‐squared measure, with maximum frequency resolution of 1 Hz and highest frequency interest of 50 Hz (Brainstorm software). The functional connectivity of SI–SIIc, SI–SIIi, and SIIc–SIIi in each individual was determined according to the following four frequency bands: theta (5–7 Hz), alpha (8–12 Hz), beta (13–30 Hz), and gamma (31–50 Hz) oscillations.

### Statistical analysis

All the data were presented as mean ± standard error of the mean (SEM). To avoid spurious type I error, nonparametric Mann–Whitney *U*‐tests were used to compare two studying groups. Associations between duration of illness and neuromagnetic signals were assessed by Spearman's correlation coefficients. A *P*‐value of < 0.05 was considered as the statistically significance.

## Results

### Verify previous SEP and SEF findings at the source level

**Figure 1:**
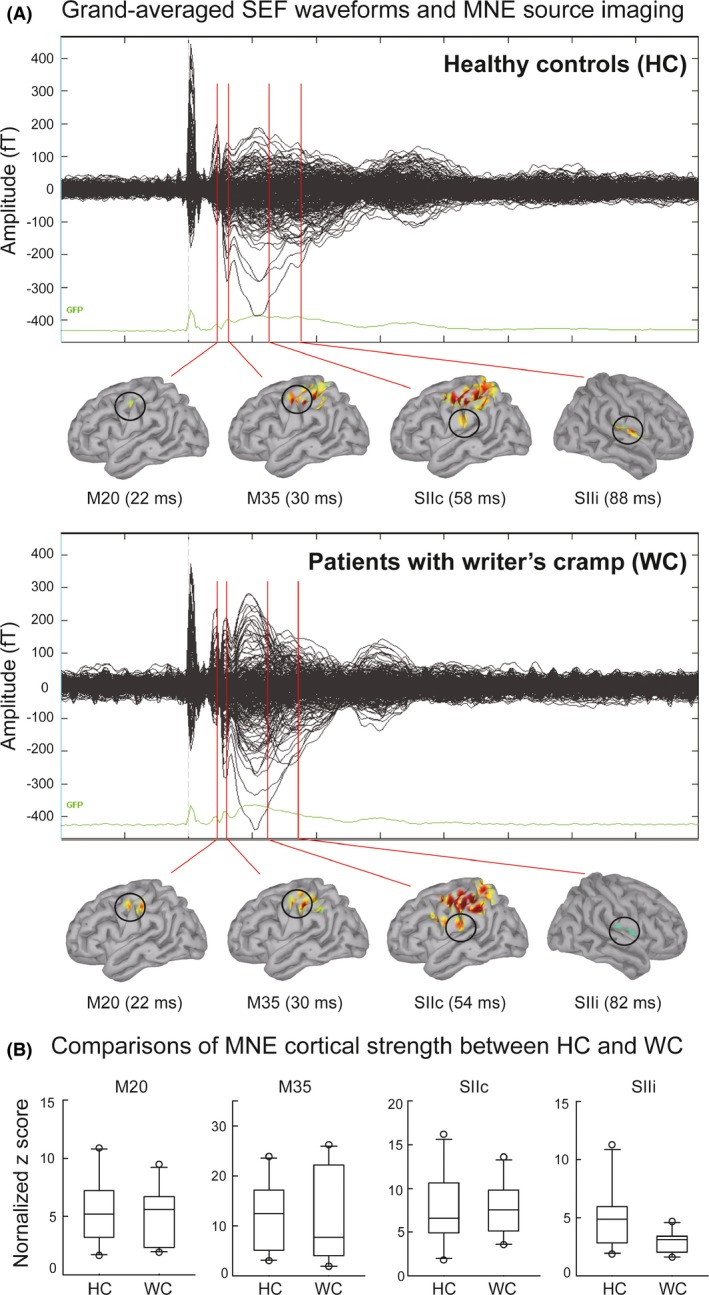
(A) Spatiotemporal dynamics of minimum norm estimate (MNE) of somatosensory‐evoked fields (SEFs) following median nerve stimulation in healthy controls (HC,* n* = 10) and patients with writer's cramp (WC,* n* = 10). The grand‐averaged MNE activation is mapped onto the Montreal Neurological Institute Colin27 template. M20 and M35 of primary somatosensory (SI) activities are located in the anterior parietal cortex. Later‐latency responses of contralateral and ipsilateral secondary somatosensory areas (SIIc and SIIi, respectively) occur in the bilateral parietal operculum with stronger activation in the SIIc than the SIIi. (B) The statistical results show no significant differences between HC and WC in terms of M20, M35, SIIc, and SIIi activation amplitudes.

Figure 1A shows the grand‐averaged SEF waveforms and the corresponding spatiotemporal cortical source maps in HC and WC groups. For the cortical MNE strength of SI (M20 and M35), SIIc, and SIIi, no significant differences were found between the two groups (Fig. 1B).

**Figure 2:**
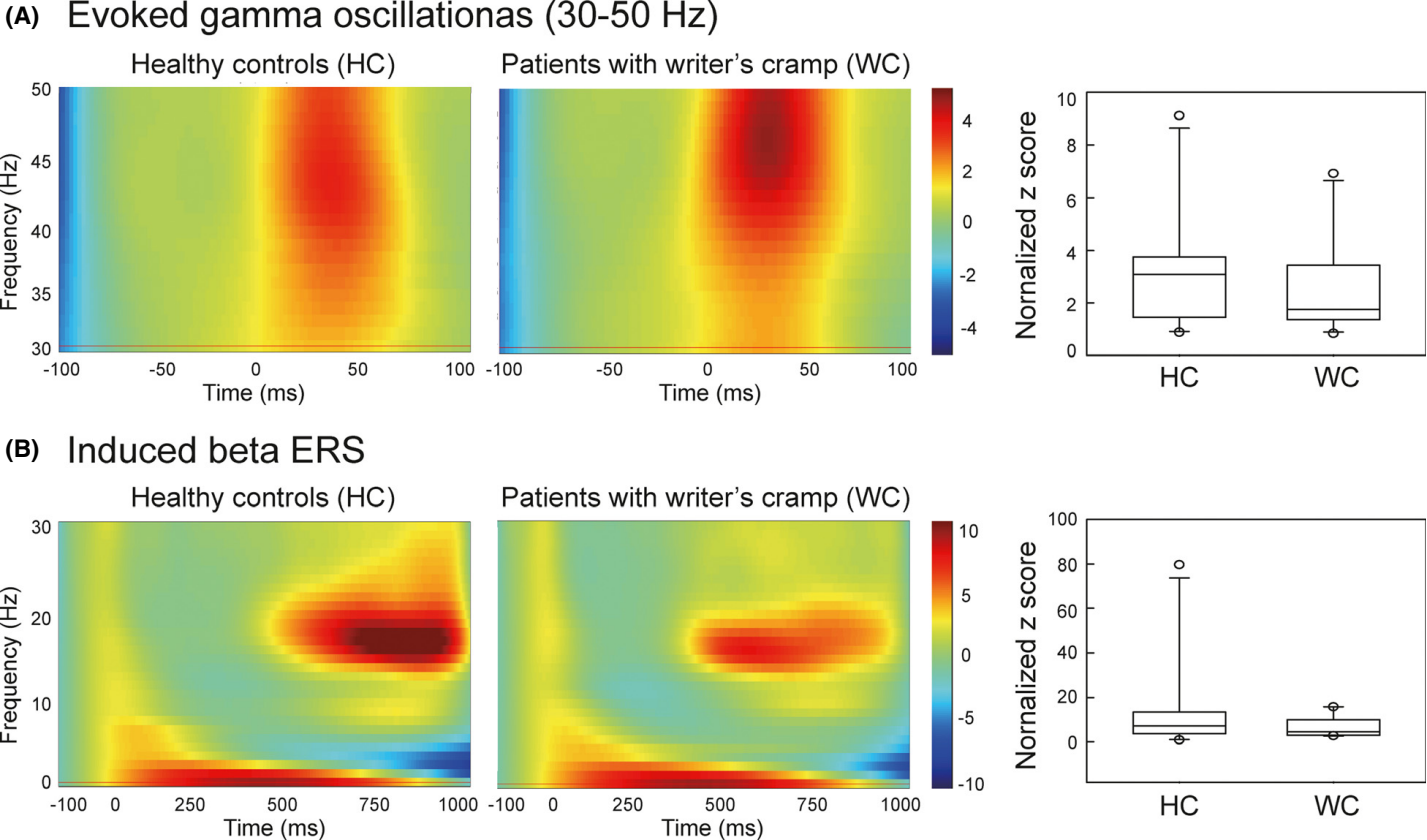
(A) Time‐frequency maps of evoked gamma oscillations in SI region. There is no difference in mean evoked gamma power (frequency range of 30 to 50 Hz and time range of 15 to 45 msec after stimulus onset). (B) Time‐frequency maps of induced beta event‐related synchronization (ERS) in SI region. Although the grand‐averaged plot shows a greater strength in the healthy controls, the statistical results exhibit no significant between‐group differences in terms of beta ERS peak amplitude.

Figure 2A shows the time‐frequency maps of evoked gamma oscillations between 30 and 50 Hz. Though the grand‐averaged plot demonstrates a stronger power in WC, the statistical analysis did not yield statistical differences in terms of mean gamma oscillatory activities. Figure 2B exhibits electrical‐induced beta ERS in each group. The peak frequencies of reactive beta bands were 19.1 ± 1.13 Hz in HC and 19.8 ± 1.23 Hz in WC. The nonparametric analysis did not show statistical significance between the groups in terms of beta ERS power.

### Primary outcome: functional connectivity

**Figure 3:**
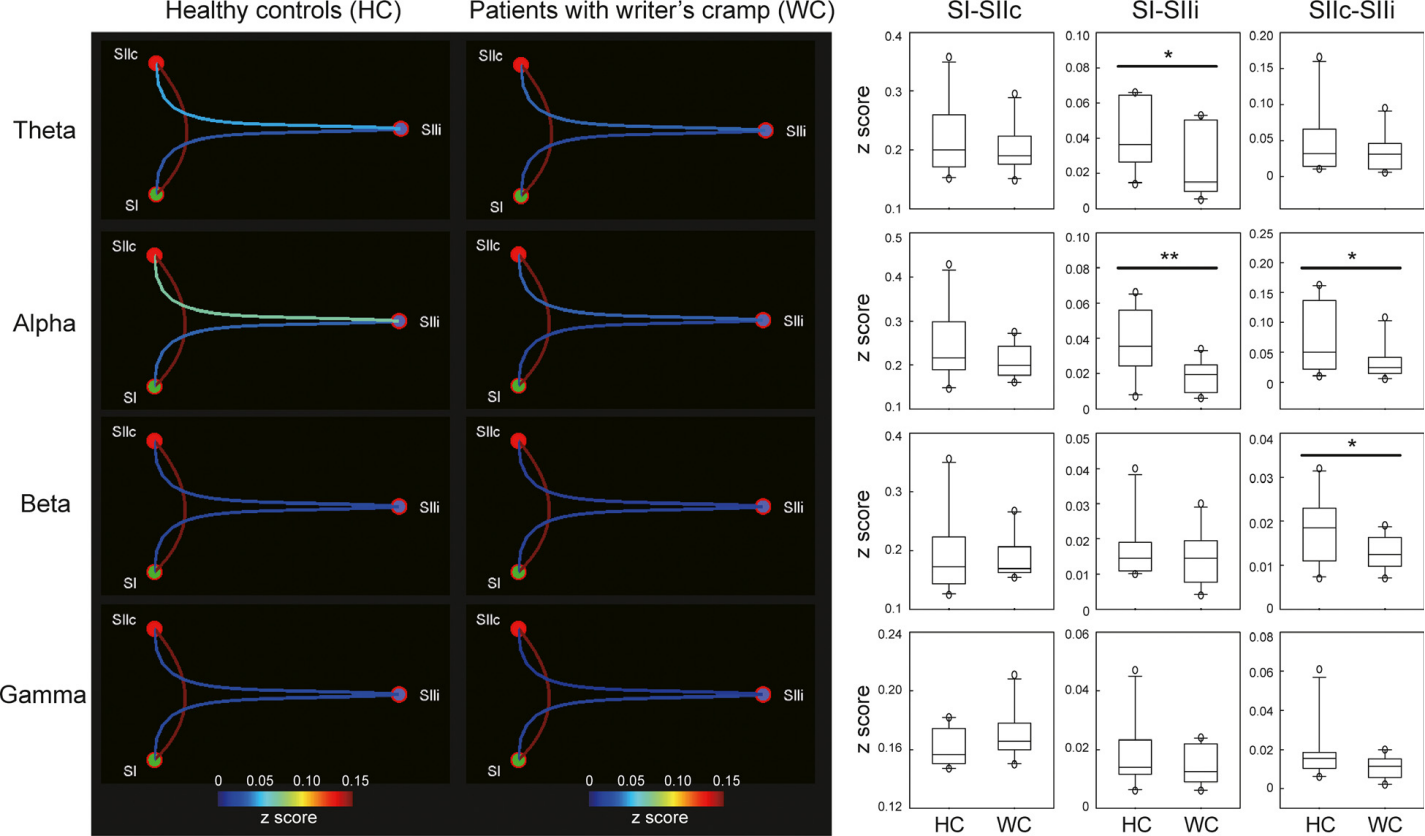
Cortical coherence of theta (5–7 Hz), alpha (8–12 Hz), beta (13–30 Hz), and gamma (31–50 Hz) oscillations in HC and WC groups. For each frequency band, the functional connectivity between SI and SIIc (SI–SIIc) is stronger than that between SI and SIIi (SI–SIIi), as well as that between SIIc and SIIi (SIIc–SIIi). Patients with WC show specific reduction in SI–SIIi coherence in the theta and alpha frequency bands, and SIIc–SIIi coherence in the alpha and beta frequency band (*p < 0.05, **p < 0.01).

We evaluated the functional connectivity among three identified ROIs (Fig. 3). Cortical coherence of SI–SIIc was in general greater than that of SI–SIIi and SIIc–SIIi for each frequency band in both HC and WC groups. Notably, the cortical coherence of SI–SIIi was strongly reduced in WC for theta (*P *=* *0.037) and alpha (*P *=* *0.006) bands. Moreover, the alpha band coherence (*P *=* *0.048) and beta band coherence (*P *=* *0.044) of SIIc–SIIi were significantly decreased in WC than in HC.

Among the aforementioned between‐group differences in terms of theta, alpha, and beta frequency bands, we did not find any statistically significant correlation between cortical coherence and disease duration.

## Discussion

To comprehensively study the neurophysiological characteristics of somatosensory system in patients with WC, we recorded MEG responses to electrical stimulation on the median nerve and analyzed these data with cortical‐based MNE and time‐frequency methods. The SI, SIIc, and SIIi source activation of WC did not differ significantly from those of matched controls. Evoked gamma frequency power and induced beta ERS strength of SI were also equivalent between the two studying groups. The most important finding was that by using functional connectivity analysis, patients with WC showed a reduced cortical coherence of SI–SIIi and SIIc–SIIi.

Early electrophysiological components of SI, such as N20 or P27 (or its magnetic counterpart of M20 or M35), refer to the fundamental somatosensory neural response to changes in afferent inputs. In consistent with previous studies (Murase et al. 2000; Dolberg et al. 2011), our results exhibited comparable SEF activities between HC and WC. Evoked gamma frequency, superimposed in time on M20 waveforms, is functionally dissociated from M20 of SI. Our current time‐frequency analysis also showed preserved gamma oscillation (30–50 Hz) energy in patients with WC, in line with previous MEG research (Cimatti et al. 2007). Interestingly, high‐frequency oscillations, usually defined as frequencies higher than 400 Hz, were found to be reduced and disorganized, suggesting desynchronized bursting of cortical neural assemblies (Cimatti et al. 2007). Owing to the insufficient sampling rates of online recordings, we were impeded to explore this component. With regards to beta ERS of SI, this study further extended previous sensor‐based analysis (Tseng et al. 2014) and indicated the nonsignificant differences in cortical power strength of beta ERS between WC and HC. Taken together, these data suggest that the altered spatial representations of digits do not necessarily affect SI cortical reactivity.

The electrophysiological studies of SII regions in patients with WC are limited. SII regions are located deep in the Sylvian fissure, and their neurophysiological function is not well‐disclosed. It has been considered that SII is involved in the higher hierarchy of somatosensory functioning, such as sensorimotor integration, perception of unified body image, and pain perception (Lin and Forss 2002). In the studies of SII reactivity in patients with focal hand dystonia, Dolberg and colleagues have reported increased amplitudes of SIIc in response to high rate (3 Hz) of tactile stimulation on the unaffected hands. In contrast, the response amplitudes of SIIc and SIIi were comparable between groups at low rate (0.5 Hz) stimulation (Dolberg et al. 2011). Our experimental design of a 3‐sec ISI (0.33 Hz), also considered as low rate stimulation, demonstrated findings with similar SII cortical activation between HC and WC.

Functional connectivity, as indexed by cortical coherence in this study, was determined by evaluating the inter‐regional oscillatory coupling in the identified 3 ROIs associated with somatosensory processing. We found statistically reduced theta and alpha cortical coherence of SI–SIIi, as well as reduced alpha and beta cortical coherence of SIIc–SIIi in patients with WC. Functional magnetic resonance imaging (MRI) studies combined with dynamic causal modeling analyses have shown serial interhemispheric connectivity (SIIc–SIIi) in the somatosensory cortex (Chung et al. 2014; Khoshnejad et al. 2014), in line with the present coherence results. However, the functional MRI data did not detect significant direct modulation between SI and SIIi. The current findings of disturbed coherence of SI–SIIi in WC might be mediated by abnormalities through indirect pathways, such as through SIIc. Inspection from the neurophysiological recordings, the earliest SI activation typically peaks at 20 msec after the stimulus onset. The longer latency SII responses usually peak at ~60 to 150 msec. This profile agrees with serial processing of somatosensory information from contralateral SI to SII, and then to the SII area of the opposite (ipsilateral) hemisphere (Allison et al. 1989; Forss et al. 1994). Taken together, these results suggest that somatosensory dysfunction may stem specifically from integration of relevant brain areas associated with somatosensation.

One might argue the effects of BTX on the somatosensory cortical responses. A previous blinded, randomized controlled study has revealed that BTX did not modulate early SEP components, such as N13, N20, or N30 (Contarino et al. 2007). To further exclude the potential influences of this treatment, the SEP or SEF recordings usually performed at least 2 to 3 months after the BTX injection (Tamura et al. 2009; Dolberg et al. 2011). In this study, only one patient received BTX treatment 3 months prior MEG recordings, and thus it might not be expected to produce apparent modification on SEF data.

In conclusion, by using MEG and time‐frequency analysis, we confirmed and extended previous studies by providing evidence of reduced functional connectivity in the somatosensory network in patients with WC. However, the locally regional somatosensory cortical responses were relatively preserved. These results imply that abnormal neural coupling among somatosensory‐associated regions may contribute to the widespread sensory or sensorimotor impairments in patients with WC.

## Conflict of Interest

The authors declare that they have no conflict of interest.
